# Prospective Whole-Genome Sequencing in Tuberculosis Outbreak Investigation, France, 2017–2018

**DOI:** 10.3201/eid2503.181124

**Published:** 2019-03

**Authors:** Charlotte Genestet, Caroline Tatai, Jean-Luc Berland, Jean-Baptiste Claude, Emilie Westeel, Elisabeth Hodille, Isabelle Fredenucci, Jean-Philippe Rasigade, Michael Ponsoda, Véronique Jacomo, Anne Vachée, Alice Gaudart, Jean-Louis Gaillard, Anne-Laure Roux, Florence Ader, Karim Tararbit, Garance Terpant, Juliet E. Bryant, Gérard Lina, Oana Dumitrescu, on behalf of the Lyon TB Study Group

**Affiliations:** Fondation Mérieux, Lyon, France (C. Genestet, J.-L. Berland, J.-B. Claude, E. Westeel, J.E. Bryant);; Centre International de Recherche en Infectiologie, Lyon (C. Genestet, J.-L. Berland, J.-B. Claude, E. Westeel, E. Hodille, J.-P. Rasigade, J.E. Bryant, G. Lina, O. Dumitrescu);; Centre de Lutte Anti Tuberculeuse, Bourg-en-Bresse, France (C. Tatai);; Hospices Civils de Lyon, Lyon (E. Hodille, I. Fredenucci, J.-P. Rasigade, F. Ader, G. Lina, O. Dumitrescu);; Laboratoires Biomnis Eurofins, Lyon (M. Ponsoda, V. Jacomo);; Centre Hospitalier Victor Provo, Roubaix, France (A. Vachée);; Centre Hospitalier Universitaire de Nice, Nice, France (A. Gaudart);; Hôpitaux Universitaires Ile de France Ouest, Ambroise Paré, Garches, France (J.-L. Gaillard, A.-L. Roux);; Agence Régionale de Santé Auvergne-Rhône-Alpes, Lyon (K. Tararbit);; Santé Publique France, Lyon (G. Terpant)

**Keywords:** Tuberculosis, Mycobacterium tuberculosis, epidemiologic monitoring, disease outbreaks, whole-genome sequencing, drug susceptibility, France, tuberculosis and other mycobacteria, bacteria, TB

## Abstract

During June 2017–April 2018, active tuberculosis with Beijing SIT1 isolates was diagnosed in 14 persons living in 4 distant cities in France. Whole-genome sequencing indicated that these patients belonged to a single transmission chain. Whole-genome sequencing–based laboratory investigations enabled prompt tracing of linked cases to improve tuberculosis control.

France is a low-prevalence country for tuberculosis (TB); mean incidence was 7.1 cases/100,000 inhabitants in 2015 (*1*). Auvergne-Rhône-Alpes is the region of France with the second highest prevalence of TB (428 cases notified in 2015). Genotyping is not yet routinely performed for all *Mycobacterium tuberculosis* isolates in France. However, prospective surveys of *M. tuberculosis* strains isolated in the Auvergne-Rhône-Alpes region have been conducted since 2008 by using systematic genotyping to detect TB transmission events and to comprehend complex relationships between certain lineages and the geographic origin of clinical cases (approved by the Comité de Protection des Personnes Sud-Est IV under no. DC-2011–1306) (*2**,**3*). Since November 2016, whole-genome sequencing (WGS) of *M. tuberculosis* isolates was implemented in the Lyon University Hospital laboratory (Lyon, France) on a routine basis to discriminate transmission clusters otherwise undetected by the spoligotyping method. We previously reported on the effect of immigration on imported *M. tuberculosis* cases in the Auvergne-Rhône-Alpes region (*3*); here, we report the rapid spread of a specific *M. tuberculosis* strain and the need for continued vigilant surveillance using advanced molecular techniques.

## The Study

During June 2017–April 2018, active TB was diagnosed in 11 persons living in the same city of Auvergne-Rhône-Alpes; all isolates belonged to Beijing SIT1 spoligotype. WGS analysis confirmed that these 11 patients were infected with the same strain, along with 3 other isolates collected in 3 different and distant cities in France, indicating a clonal outbreak.

**Figure 1:**
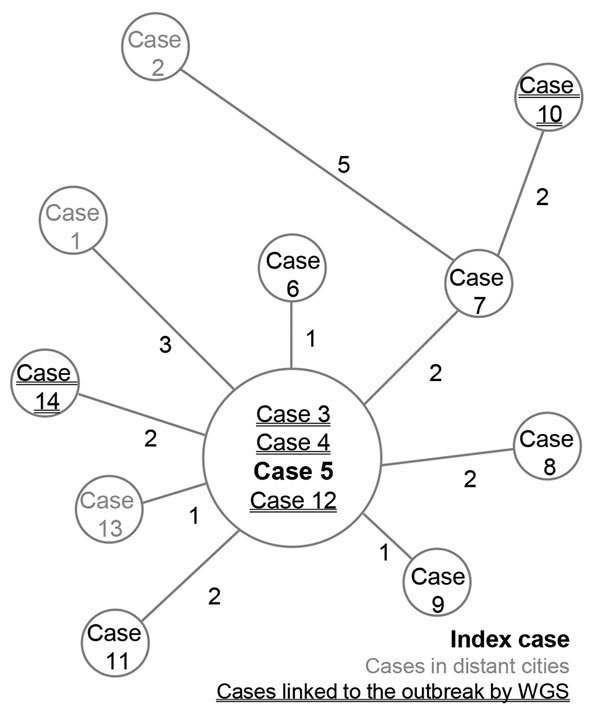
Minimum-spanning tree of single-nucleotide polymorphism differences from 14 *Mycobacterium tuberculosis* outbreak isolates, France, 2017–2018. Cases were numbered according to sampling chronology. Sizes of circles are proportional to the number of isolates with identical genomes; numbers adjacent to lines indicate number of single-nucleotide polymorphism differences between each node. WGS, whole-genome sequencing.

During June–September 2017, three cases of active TB were diagnosed from the same city in the Auvergne-Rhône-Alpes region. Patients belonged to 2 families without previously known relationships. One of the 3 patients (index case-patient [chronological case-patient 5]) had a 2-year history of confirmed TB; this patient originated from Cape Verde and reported frequent recent travel there. Direct microscopic examination of sputum showed very high titers of acid-fast bacilli, implying a high risk for onward *M. tuberculosis* transmission. In the other 2 case-patients (chronological case-patients 3 and 4), TB was newly diagnosed; they had no history of or suspicion for infection, but both resided in the same household. Isolates from all 3 cases were susceptible to standard first-line anti-TB drugs, and spoligotyping revealed that all belonged to Beijing SIT1 lineage. In parallel with conventional membrane-based spoligotyping, WGS was performed to ascertain whether it was the same strain (Appendix). WGS analysis indicated that the strains were identical, meaning 0 single-nucleotide polymorphism (SNP) distance (Figure 1).

Investigations and interviews conducted by the TB control center later revealed that the 2 families did have a connection, through frequent contacts between case-patient 4 and the index case-patient (case-patient 5). These results led the TB control center to widen the circle of investigations beyond household and frequent contacts and to expand the investigation to occasional contacts (including persons from other geographic regions) and persons attending the public places frequented by the index case-patient.

During September 2017–April 2018, extended contact tracing identified 5 additional cases of active TB within the index city and 3 epidemiologically linked cases in distant areas (Île-de-France, Hauts-de-France, and Provence-Alpes-Côte d’Azur). WGS analysis confirmed that all patients belonged to a single transmission chain, supporting a rapid dissemination of this epidemic strain in France. TB had been diagnosed in 1 of the in-contact case-patients as early as 2015; case-patient 1 and the index case-patient lived together in 2015 in Île-de-France, during which time the transmission event was hypothesized to have occurred. These findings led to an extended investigation and communication outreach to general practitioners in the index city. General practitioners were informed of the outbreak and prompted to prioritize performing TB detection in patients with respiratory symptoms suspected to be TB. Consequently, during early 2018, active TB caused by the Beijing SIT1 epidemic strain was diagnosed in 3 previously non–epidemiologically linked patients. Subsequent investigations showed previously missed occasional contacts between the index case-patient and these 3 TB case-patients.

Out of the 14 case-patients identified, 9 (64%) infected by the Beijing strain were household or frequent contacts, and 5 (36%) were occasional contacts (Table). Five case-patients were children. Pulmonary and extrapulmonary disease developed in 4 case-patients; in 2 of those, meningitis, a severe form of TB associated with high rates of death and disability, developed.

**Table:** Summary of contact tracing and description of cases in a tuberculosis outbreak, France, 2017–2018

Case-patient	Date of sample collection	Contact with index case-patient	Age	Origin	Diagnosis region	TB infection site
1	3rd trimester 2015	Household	Adult	Non–France-born	Île-de-France	Lung
2	1st trimester 2017	Occasional	Adult	Non–France-born	Hauts-de-France	Lung
3	2nd trimester 2017	Household*	Child	France-born	Auvergne-Rhône-Alpes	Lung, meningitis
4	3rd trimester 2017	Frequent	Adult	France-born	Auvergne-Rhône-Alpes	Lung
5†	3rd trimester 2017	Household	Adult	Non–France-born	Auvergne-Rhône-Alpes	Lung
6	3rd trimester 2017	Household	Child	France-born	Auvergne-Rhône-Alpes	Lung, adenitis
7	4th trimester 2017	Household	Child	France-born	Auvergne-Rhône-Alpes	Lung
8	4th trimester 2017	Household	Child	France-born	Auvergne-Rhône-Alpes	Lung
9	4th trimester 2017	Frequent	Adult	Non–France-born	Auvergne-Rhône-Alpes	Lung
10	4th trimester 2017	Occasional	Adult	Non–France-born	Auvergne-Rhône-Alpes	Lung
11	1st trimester 2018	Frequent	Adult	Non–France-born	Auvergne-Rhône-Alpes	Lung
12	1st trimester 2018	Occasional	Adult	Non–France-born	Auvergne-Rhône-Alpes	Lung, adenitis
13	1st trimester 2018	Occasional	Child	France-born	Provence-Alpes-Côte d'Azur	Lung, meningitis
14	1st trimester 2018	Occasional	Adult	France-born	Auvergne-Rhône-Alpes	Lung

*Household from case-patient 4.†Index case-patient.

Among the 14 cases identified, 5 (36%) were linked to this outbreak through laboratory investigations. WGS analysis enabled us to link strains from 2 initially independent contact tracing investigations and relate 5 cases with no previously identified epidemiologic link. Pairwise SNP distance comparison of the isolates revealed a maximum of 7 SNPs separating the index case-patient from the other 13 case-patients and a maximum of 10 SNPs separating other cases (Figure 1). The median SNP difference between cases with a verified epidemiologic link was 2 (range 0–5). This finding is consistent with previous studies in which a 12-SNP threshold excluded transmission (*4*). We compared the cluster we report with other Beijing SIT1 strains isolated in 2017 in our region: pairwise SNP comparison ranged from 140- to 330-SNP distance. This cluster is therefore unrelated to other Beijing SIT1 contemporarily and regionally circulating strains. According to the Shitikov scheme (*5*), those strains belong to the Central Asia group.

## Conclusions

**Figure 2:**
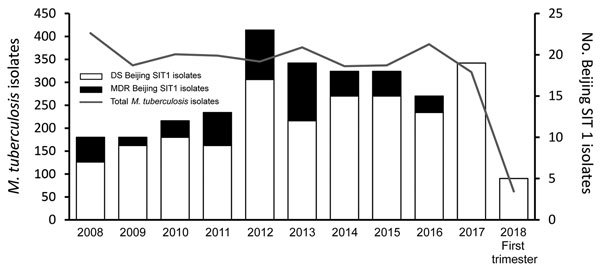
Prevalence of tuberculosis cases and *Mycobacterium tuberculosis* Beijing SIT1 genotype, Auvergne-Rhône-Alpes, France, 2008–2018. DS, drug-sensitive; MDR, multidrug-resistant.

The Beijing strain family, regardless of the antimicrobial susceptibility profile, has been involved in many TB outbreaks, including in Europe (*6**–**10*). Some studies suggest that the epidemic success of the modern Beijing strains might be linked to increased virulence, higher transmission rates, or both, but findings have not been consistent (*11**–**13*). In the Auvergne-Rhône-Alpes region, Beijing strain accounts for 2.5%–8.1% of all *M. tuberculosis* isolates (average 4.9%) (Figure 2), depending on the year. The increased incidence of Beijing genotype during 2011–2013 (i.e., up to 7.3% in 2012) was related to the migratory influx of patients with multidrug-resistant TB from Eastern Europe and Asia (*14*). The 8.1% increased incidence of Beijing genotype from the second half of 2017 and the first 4 months of 2018 was due to the outbreak we describe.

WGS led to the rapid identification of this transmission event. Real-time feedback between the microbiology laboratory and the TB control center prompted further contact investigations and identification of additional epidemiologically linked cases.

This report highlights how, beyond the traditional contact tracing investigations, molecular *M. tuberculosis* typing enabled detection of otherwise unsuspected transmission and therefore identification of extended clusters. This finding is important because it is not uncommon that TB patients do not cooperate well with interviews (*15*); even in low-prevalence countries, stigma associated with TB diagnosis often hampers adherence of patients to screening. Noncomplying patients do not disclose exhaustive lists of contacts; as shown in this study, case-patients 4 and 5 were found to have frequent contact only through the second contact-tracing interview. In this respect, robust microbiological evidence of transmission may encourage TB control units to perform second interviews and thus increase the chances to document more transmission events. Our observations underscore the added value of molecular epidemiologic tools to prompt TB contact investigations for better detection and disease control.

Appendix Additional information about whole-genome sequencing in a tuberculosis outbreak investigation, France, 2017–2018.
