# Supplementary material for: Prospective Whole-Genome Sequencing in Tuberculosis Outbreak Investigation, France, 2017–2018
Source: Emerg Infect Dis. 2019 Mar;25(3):589–92. doi: 10.3201/eid2503.181124 (PMC6390740; doi:10.3201/eid2503.181124)
Supplement: Appendix — Additional information about whole-genome sequencing in a tuberculosis outbreak investigation, France, 2017–2018. [file 18-1124-Techapp-s1.pdf]

Article DOI: https://doi.org/10.3201/eid2503.181124

Prospective Whole-Genome Sequencing in Tuberculosis Outbreak Investigation, France, 2017–2018

Appendix

Whole-Genome Sequencing

Genomic DNA was purified from cleared lysate using a QIAamp DNA mini Kit (QIAGEN, Courtaboeuf, France). DNA libraries were prepared with Nextera XT kit (Illumina, San Diego, CA, USA). Samples were sequenced on Miseq system (Illumina) at the University of Lyon facility (Lyon, France), to produce 75–300 base-pair paired-end reads. These reads were mapped with BOWTIE2 (*1*) to the H37Rv reference genome (Genbank NC000962.2). Variant calling was made with SAMtools mpileup (*2*): a valid nucleotide variant was called if the position was covered by at least 10 reads depth and supported by a minimum rate threshold of 50%. Region with repetitive or similar sequences were excluded, i.e., PE, PPE, PKS, PPS, ESX. The WGS genome coverage ranges 96.8%–98.2%, average depth 44x to 127x.

To challenge the performance of the pipeline and measure the number of SNPs threshold between isolates with epidemiologic links, we ran the archives from Walker et al., 2013 (*3*): a threshold of 12 SNPs was retained as the limit to exclude a cross-transmission.

Sequences have been submitted to European Nucleotide Archive (ENA) under accession no. ERP111099.

References

1. Langmead B, Salzberg SL. Fast gapped-read alignment with Bowtie 2. Nat Methods. 2012;9:357–9. PubMed http://dx.doi.org/10.1038/nmeth.1923

2. Li H, Handsaker B, Wysoker A, Fennell T, Ruan J, Homer N, et al.; 1000 Genome Project Data Processing Subgroup. The Sequence Alignment/Map format and SAMtools. Bioinformatics. 2009;25:2078–9. PubMed http://dx.doi.org/10.1093/bioinformatics/btp352

3. Walker TM, Ip CL, Harrell RH, Evans JT, Kapatai G, Dedicoat MJ, et al. Whole-genome sequencing to delineate *Mycobacterium tuberculosis* outbreaks: a retrospective observational study. Lancet Infect Dis. 2013;13:137–46. PubMed http://dx.doi.org/10.1016/S1473-3099(12)70277-3
